# Analysis of Gene Expression of miRNA-106b-5p and *TRAIL* in the Apoptosis Pathway in Gastric Cancer

**DOI:** 10.3390/genes11040393

**Published:** 2020-04-05

**Authors:** Jéssica Pereira, Mônica Santos, Roger Delabio, Mônica Barbosa, Marília Smith, Spencer Payão, Lucas Rasmussen

**Affiliations:** 1 Marilia Medical School (FAMEMA), Marília, São Paulo 17519-030, Brazil; jessica.np14@hotmail.com (J.P.); monica.pezenatto@hotmail.com (M.S.); roger@famema.br (R.D.); slmpayao@hotmail.com (S.P.); 2 Department of Biosciences and Technology of Institute of Tropical Pathology and Public Health, Federal University of Goias (UFG), Goiânia, Goiás 74605-050, Brazil; santiago@ufg.br; 3 Department of Morphology and Genetics, Escola Paulista de Medicina, Federal University of Sao Paulo (UNIFESP), São Paulo 04023-062, Brazil; macsmith@unifesp.br

**Keywords:** *Helicobacter pylori*, gastric cancer, apoptosis, *TRAIL*, microRNA-106b-5p

## Abstract

*Helicobacter pylori* (*H. pylori*) is one of the main causes of gastric gancer. *TNF-related apoptosis-inducing ligand (TRAIL)* is a protein able to promote apoptosis in cancer cells, however not in gastric cancer, which presents resistance to apoptosis via *TRAIL*. It is believed that MicroRNA-106b-5p might be involved in this resistance, although its role in Gastric Cancer is unclear. We aimed to determine the expression of microRNA-106b-5p and *TRAIL* in patients with gastric diseases, infected by *H. pylori*, and understand the relationship between these genes and their role in apoptosis and the gastric cancer pathways. *H. pylori* was detected by PCR, gene expression analysis was performed by real-time-qPCR, and bioinformatics analysis was performed using the Kyoto Encyclopedia of Genes and Genomes (KEGG) and Cytoscape software. A total of 244 patients were divided into groups (Control, Gastritis, and Cancer); *H. pylori* was detected in 42.2% of the samples. The cancer group had a poor expression of *TRAIL* (*p* < 0.0001) and overexpression of microRNA-106b-5p (*p* = 0.0005), however, our results confirmed that these genes are not directly related to each other although both are apoptosis-related regulators. Our results also indicated that *H. pylori* decreases microRNA-106b-5p expression and that this is a carcinogenic bacterium responsible for gastric diseases.

## 1. Introduction

Gastric cancer is one of the most commonly diagnosed types of cancer—the fifth most frequent worldwide, and the third highest cause of cancer-related death—and for many years, it has been considered a global health problem [1,2,3,4]. Gastric cancer is a multifactorial disease, depending on factors such as patients’ lifestyle, diet, and genetic characteristics [5]. *Helicobacter pylori* (*H. pylori*) has also been considered one of the main causes of gastric diseases, especially gastric cancer, considering that this Gram-negative bacterium successfully colonizes the stomach promoting intense inflammation and deregulating apoptosis process [6,7,8].

Apoptosis is a mechanism of programmed cell death; it is essential for the maintenance of gastric tissue by controlling cell proliferation, and is considered a defense mechanism against neoplastic processes [9,10,11]. The apoptosis process can be activated by two pathways: the mitochondrial pathway (intrinsic pathway) and the death receptor pathway (extrinsic pathway) [10]. The latter pathway depends on the link between specific ligands that leads to cell death [12].

*TNF-related apoptosis-inducing ligand (TRAIL)* is a transmembrane protein that promotes apoptosis by binding to its death receptors *TRAIL-R1 (DR4)* and *TRAIL-R2 (DR5)*, targeting cancer cells and sparing healthy and normal cells [13,14,15]. *TRAIL* selectivity to cancer cells is justified by the high expression of *DR4* and *DR5* receptors in these cells, which facilitates *TRAIL* binding and the consequent activation of apoptosis [16]. However, some types of cancer have presented resistance to apoptosis via *TRAIL*, including gastric cancer [17,18].

MicroRNAs (miRNAs), small and non-coding RNAs, are molecules that bind to the 3‘-untranslated region (3′-UTR) of their corresponding target mRNAs and control the expression of specific genes [19,20]. These molecules are made up of about 20 nucleotides and participate in many different biological processes, such as cell proliferation, differentiation, and apoptosis; moreover, miRNAs are involved in the onset, progression, and metastasis of cancer [19,21]. Located on chromosome 7, microRNA-106b-5p (miR-106b-5p) has been considered an onco-miRNA due to its increased expression in different cancer types like hepatocellular carcinoma, breast cancer, osteosarcoma, and gastric cancer. It also plays a functional role in malignancy-associated processes including cell proliferation and migration [22,23,24,25].

Considering the above, this work aimed to determine the expression of miR-106b-5p and *TRAIL* in patients with gastric disease, including patients with gastric cancer, infected or not by *H. pylori*, We took miR-106b-5p as a focus of this study since only a few works correlated this gene to gastric cancer and the *TRAIL*, including the presence of *H. pylori* as a parameter to the analysis.

## 2. Materials and Methods

### 2.1. Patients and Tissue Samples

This study analyzed samples from 244 patients of both genders and over 18 years old. Three gastric biopsy samples were obtained from each patient: one for histopathological analysis to determine groups, one for DNA extraction, and one for RNA extraction. Samples were collected from patients who presented dyspeptic symptoms and were submitted to upper endoscopy, and from patients with gastric cancer submitted to surgery. Histopathological analysis was performed according to Sydney and Lauren’s criteria [5,26] to separate the patients into groups: Control (patients with healthy gastric mucosa), Gastritis, and Cancer. Details are described in Table 1.

All the patients involved in this research signed a consent form. Patients were excluded from this study who had received antimicrobial, anti-inflammatory, and chemotherapy within 30 days of the medical procedure. This research was approved by the Research Ethics Committee (number 1.119.830).

The samples from control patients and patients with gastritis were collected at the State Hospital of Bauru (HEB) and the Gastroenterology Center of the Clinical Hospital of Marilia Medical School (HC). The samples from patients with gastric cancer were obtained in collaboration with the Federal University of Sao Paulo (UNIFESP) and the Federal University of Goias (UFG).

### 2.2. DNA Extraction and Helicobacter Pylori Detection

DNA extraction was performed using QIAamp^®^ DNA Mini Kit (Qiagen, Hilden, Germany, cat. No 51304,) according to the manufacturer’s instructions. *H. pylori* was diagnosed employing polymerase chain reaction (PCR) using the *Hpx1* (CTGGAGARACTAAGYCCTCC) and *Hpx2* (GAGGAATACTCATTGCGAAGGCGA) oligonucleotides, under conditions of 40 cycles: 94 °C, 1 min; 59 °C, 1 min, and 72 °C, 1 min. The diagnosis of *H. pylori* was obtained by electrophoresis, through which a fragment of 150 bp was visualized by agarose gel electrophoresis 2.5%, stained with ethidium bromide, and viewed and photographed in a transilluminator on the α Imager 2200 image capture system (α Innotech Corporation) [27].

### 2.3. RNA Extraction, cDNA Synthesis, and Real-Time Quantitative PCR (qPCR) Gene Expression Analysis

The samples collected for RNA extraction were stored in RNAlater^®^ Tissue Collection (Ambion, Woodlands, TX, USA) at −20 °C. RNA extraction was performed using miRNeasy*^®^* Mini Kit 50 (Qiagen, cat. No 217004) according to the manufacturer’s protocol. The quality of RNA was confirmed using a Nanodrop ND-1000 (Thermo Fisher Scientific, Waltham, MA, USA).

Complementary DNA (cDNA) synthesis from total RNA was performed with the High-capacity cDNA Reverse Transcription Kit (Applied Biosystems™, Waltham, MA, USA) for expression analysis of *TRAIL*; the cDNA synthesis for microRNA analysis was performed using the TaqMan^®^ MicroRNA Reverse Transcription Kit (Applied Biosystems™, USA). Both procedures were performed according to the manufacturer’s instructions.

The expression was determined by the ABI Prism 7500 Fast Sequence Detection System using TaqMan assays (Applied Biosystems). The qPCR used *TRAIL* (Hs00366278_m1) and hsa-miR-106b-5p (000442) as target genes. The reference genes for normalization were *UBC* (Hs00221499_m1) and *TBP* (Hs00187332_m1) for *TRAIL,* and for miR-106b-5p, RNU6B (Hs001093) and RNU48 (Hs001006) were employed. The relative quantification of the expression was calculated using the 2^−ΔΔCt^ method [28].

### 2.4. Bioinformatics Analysis

#### 2.4.1. Identification of miRNAs and Target Genes in Apoptosis

In order to clarify the role played by *TRAIL* and miR-106b-5p genes in the apoptosis pathway, bioinformatics analysis was performed using the Kyoto Encyclopedia of Genes and Genomes (KEGG) (https://www.genome.jp/kegg/) to first identify the genes involved in the apoptosis pathway (pathway: map04210). Then, considering only the apoptosis pathway, we used miRWalk online databases (http://zmf.umm.uni-heidelberg.de/apps/zmf/mirwalk2/index.html) to determine the target miR-106b-5p genes and the miRNAs that target the *TRAIL* gene. The interaction network was constructed using the Cytoscape software (version 3.7.2).

#### 2.4.2. Screening for Differentially Expressed Genes (DEGs)

An in silico analysis was also performed to identify differentially expressed genes (DEGs) using two gene expression profiles between gastric cancer and normal gastric tissue samples from the GEO database ((https://www.ncbi. nlm.nih.gov/geo/) to confirm our preliminary results.

Using the keyword “apoptosis and gastric cancer” to search on the GEO Datasets database (https://www.ncbi.nlm.nih.gov/gds/?term=Apoptosis+and+gastric+cancer), a total of 27 series about human gastric cancer and apoptosis were retrieved from the database. After a careful review, two gene expression profiles on the GEO Datasets database (Accession: GSE103236 and Accession: GSE33651) were downloaded.

The DEGs analysis was performed using the GEO2R online analysis tool (http://www.ncbi.nlm.nih.gov/geo/geo2r), and the corrected *p*-values and |logFC| were calculated and used for the elaboration of volcano plot to determine differences in expression. The Venn diagram analysis was carried out for the intersection part via Funrich software (http://funrich.org/).

### 2.5. Statistical Analysis

The data were analyzed using GraphPad Prism 5. One-way analysis of variance (ANOVA), two-tailed Student’s *t*-test, and Fisher’s Exact test were employed. Significant results were considered those where *p* ≤ 0.05.

## 3. Results

### 3.1. Helicobacter Pylori Detection

**Figure 1:**
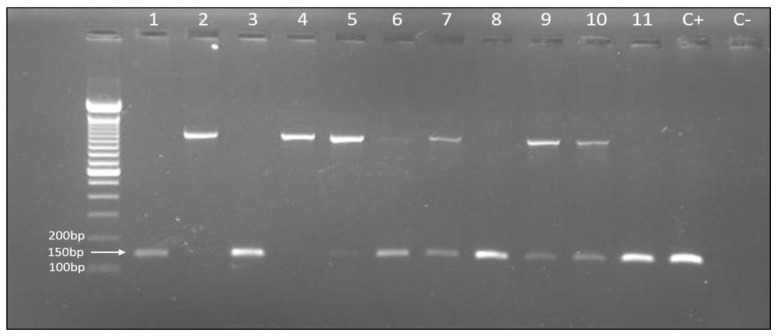
Two percent agarose gel stained with ethidium bromide showing *H. pylori* PCR products (150 bp) used to identify *H. pylori* in gastric biopsy samples from patients with normal gastric mucosa (1 to 3), gastritis (4 to 7), and gastric cancer (8 to 11). Water was used as negative control; as positive control, *H. pylori* DNA from culture was used. The band of approximately 1200 bp is a non-specific band, not associated with *H. pylori.*

After separating the groups by histopathological analysis, the first analysis performed was PCR for *H. pylori* detection. Using specific primers described in the Materials and Methods section, a 150 bp fragment corresponding to the bacterium was identified. *H. pylori* was detected in 42.2% of the samples. Our results indicate that this bacterium is related to the occurrence of gastric diseases including gastric cancer, considering that it was prevalent in the groups of patients with gastric injury and rare in healthy patients. Figure 1 illustrates the result of the electrophoresis (extra figures are available at Supplementary Materials) and Table 2 describes the details of positive samples for *H. pylori*.

### 3.2. Analysis of TRAIL and microRNA-106b-5p Gene Expression

For both *TRAIL* and miR-106b-5p, the expression levels were analyzed in two parts: first, the presence of *Helicobacter pylori* was not considered; thus, the Control, Gastritis, and Cancer groups consist of patients both infected and uninfected by the bacterium. Then, the second analysis was performed considering the presence of the bacterium; therefore, each group was divided into positive (Pos.) and negative (Neg.).

**Figure 2:**
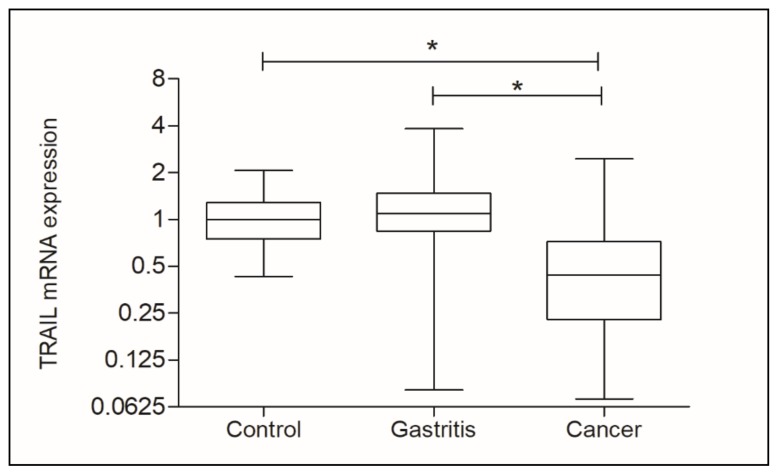
Analysis of *TNF-related apoptosis-inducing ligand* (*TRAIL*) gene expression in Control, Gastritis, and Cancer groups, considering patients that were positive and negative for *H. pylori* infection in all groups. * statistically significant.

A statistically significant difference (*p* < 0.0001) was found between the three main groups of this study (Control, Gastritis, and cancer) in relation to *TRAIL* gene expression. Figure 2 shows that *TRAIL* expression was significantly decreased in the Cancer group (RQ mean: 0.4400) compared to the Control group (RQ mean: 1.000, *p* < 0.0001) and the Gastritis group (RQ mean: 1.090, *p* < 0.0001).

**Figure 3:**
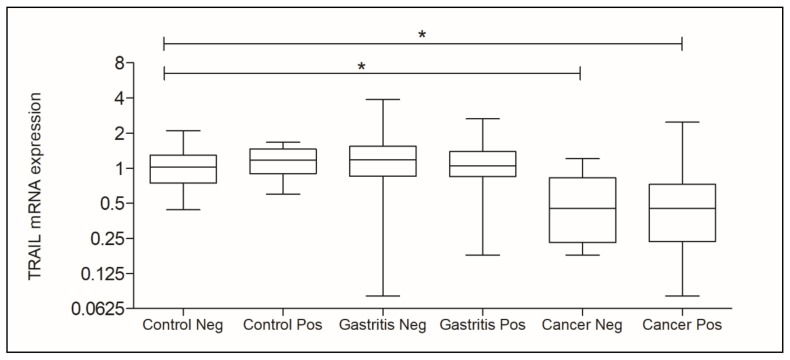
Analysis of *TRAIL* gene expression for the presence of *H. pylori* in Control, Gastritis, and Cancer groups. Legend: Neg.: Negative; Pos.: Positive. *statistically significant.

When considering the presence of *H. pylori*, each new group was compared with the Control Negative group (Control Neg.). There was a statistically significant difference when comparing the Control Neg. group (RQ mean: 1.020) to Cancer Neg. group (RQ mean: 0.4500, *p* = 0.0003) and the same Control Neg. group to Cancer Pos. group (RQ mean: 0.4500, *p* < 0.0001) as indicated in Figure 3.

Our results demonstrated that *H. pylori* does not interfere in the *TRAIL* gene expression, since the analysis did not present statistically significant differences when comparing the positive and negative groups. According to Figure 3, it is possible to notice that the expression levels of *TRAIL* are similar when the groups were compared to each other (Control Neg vs. Control Pos; Gastritis Neg vs. Gastritis Pos and Cancer Neg vs. Cancer Pos).

MiR-106b-5p gene expression was statistically different between the Control, Gastritis, and Cancer groups without considering the presence of *H. pylori* (*p* = 0.0005). The results of the comparisons between Control vs. Gastritis, Control vs. Cancer, and Gastritis vs. Cancer were all statistically significant as detailed in Table 2.

**Figure 4:**
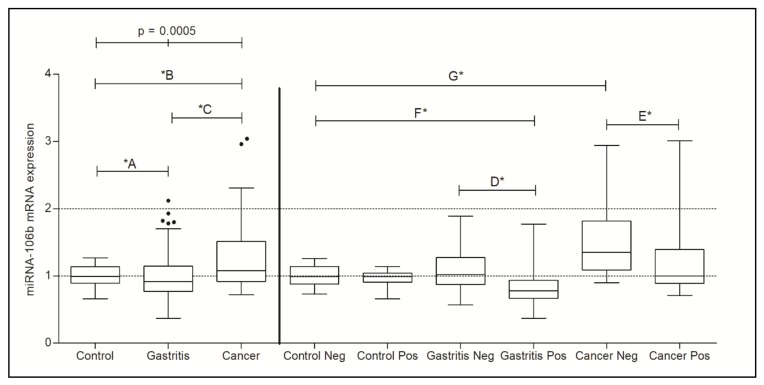
Analysis of miR-106b-5p gene expression not considering the presence of *H. pylori* in Control, Gastritis, and Cancer groups as well as analysis of miR-106b-5p gene expression with the presence of *H. pylori*. Legend: Neg.: Negative; Pos.: Positive. * statistically significant and *p*-values showed in Table 3.

The presence of *H. pylori* was considered in the second part of the analysis; all groups were compared to Control Neg. group and the results for RQ mean and *p*-value are in Table 3. Figure 4 shows miR-106b-5p expression levels in all groups considered in this study.

When the groups of Gastritis Pos. were compared with Gastritis Neg and the groups of Cancer were compared to each other, both Gastritis Pos. (*p* < 0.0001 *D) and Cancer Pos. (*p* = 0.0192 *E) presented a decrease in miR-106b-5p expression and it seems that it is due to the presence of *H. pylori*, for this bacterium seems to be able to inhibit this miRNA expression.

Our results did not show any statistically significant difference when comparing the gene expression of *TRAIL* and miR-106b-5p with the parameters age and sex.

### 3.3. Bioinformatics Analysis and Interaction Network in the Apoptosis Pathway Considering TRAIL and miR-106b-5p Genes

It is known that both *TRAIL* and miR-106b-5p are related to the apoptosis process. In this work, we attempted to understand the relation between these genes and their function in the apoptosis pathway; therefore, bioinformatics analysis was performed to elucidate the interactions.

**Figure 5:**
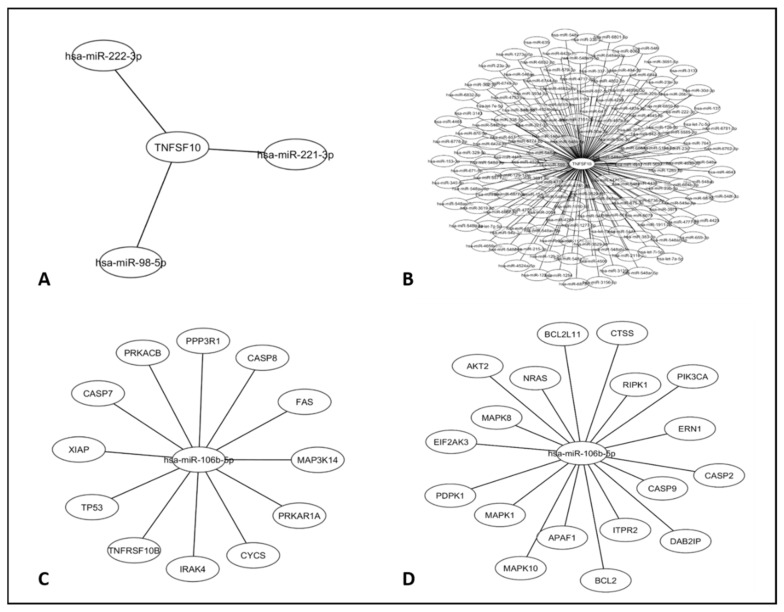
Cytoscape analysis considering *TRAIL* and miR-106b-5p in the apoptosis pathway. (**A**) Validated miRNAs that interact with the *TRAIL* (*TNFSF10*) gene. (**B**) Predicted miRNAs that have *TRAIL* (*TNFSF10*) as target gene. (**C**) Validated genes controlled by miR-106b-5p. (**D**) Predicted genes controlled by miR-106b-5p.

First, we took *TRAIL* (*TNFSF10*) as a reference gene in the apoptosis pathway and searched for miRNAs that had this gene as target. Figure 5A shows the miRNAs that were validated and Figure 5B shows the predicted ones. In this analysis, miR-106b-5p was not among the results of direct interaction with the *TRAIL* gene.

The second analysis was performed taking the miR-106b-5p as a reference gene and we searched for genes that were affected by this miRNA in the apoptosis pathway. Figure 5C shows the validated genes and Figure 5D shows the predicted ones. *TRAIL* is not one of the target genes of miR-106b-5p; however, this miRNA has other genes as target that make up this apoptosis pathway, such as *Fas*, *Casp8*, and *Casp7*, indicating that this miRNA, in fact, is able to control cell death.

Our results confirm that *TRAIL* and miR-106b-5p are apoptosis-related regulation, but they are not directly related to each other. *TRAIL* starts the apoptosis process by binding to its receptors and other genes continue the process inside the cell to reach the cell death, and these genes can be controlled by miR-106b-5p.

### 3.4. Identification of Differentially Expressed Genes (DEGs)

**Figure 6:**
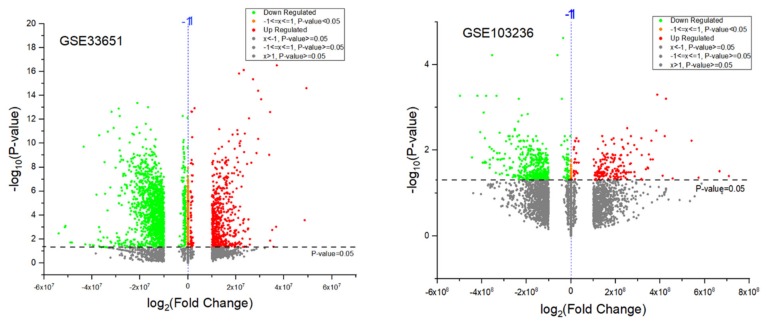
The Volcano plots (GSE33651 and GSE103236) showed the differentially expressed genes (DEGs) between gastric cancer samples and normal samples.

When the GSE103236 dataset was screened by GEO2R and then submitted to analysis, we verified that 199 genes were upregulated, and 532 genes were downregulated. Considering the GSE33651, 608 genes were found upregulated, and 1645 genes downregulated (Figure 6).

It is important to highlight that, considering the 1645 genes found in the GSE33651 dataset with a decrease of expression, the *TRAIL* gene was located at position 268, confirming our results for a decrease in the expression of this gene. On the other hand, for the *TRAIL* gene, although appearing in the GSE103236 dataset, an expression differential was not verified.

**Figure 7:**
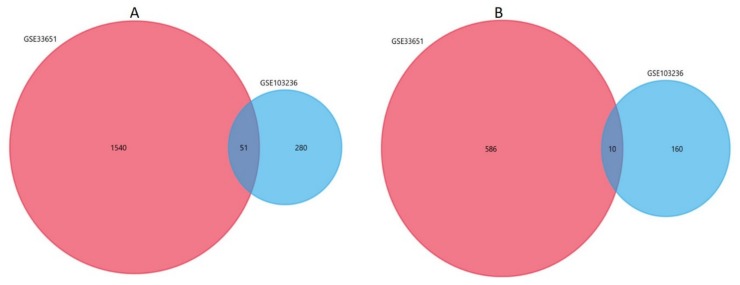
Venn diagrams representing the overlaps between GSE33651 and GSE103236 datasets. (**A**) Venn diagrams illustrating the overlap of downregulated genes. (**B**) Venn diagrams illustrating the overlap of upregulated genes.

We generated a list of potential candidate genes that are differentially expressed in both datasets using Venn analysis (Figure 7). So, 61 DEGs were significantly differentially expressed between all two datasets, of which 10 were significantly upregulated genes and 51 were downregulated (Table 4).

## 4. Discussion

*H. pylori* infects the stomach of over 50% of the world’s population [6]. Since its discovery in 1983 [29], scientists have demonstrated that *H. pylori* is related to the occurrence of different types of gastric diseases including gastric cancer [30] through several mechanisms that this bacterium developed over the years [31]. In this study, *H. pylori* was identified in 42.2% of the 244 analyzed samples and its prevalence was 73.1% in the Cancer group. When compared to the other groups, this relevant result (*p* > 0.0001) confirms that *H. pylori* is, in fact, responsible for gastric diseases and is a carcinogenic bacterium. These findings are in agreement with other studies [32,33,34].

*TRAIL* is a pro-apoptotic protein that starts the apoptosis process by binding to its death receptors *DR4* and *DR5*, targeting tumor cells without attacking healthy cells [35,36,37]. For this reason, *TRAIL* has been considered a promising cancer therapy [16]; however, some studies have shown that gastric cancer cells are resistant to this apoptosis pathway [13,17,18,38].

Our results for *TRAIL* expression found that this gene was poorly expressed in the Cancer group compared to Gastritis and Control groups. We suggest that this low expression of *TRAIL* is one of the causes for cancer, because a poorly expressed mRNA translates into low protein, which, in turn, is found in low levels, insufficient to act as expected for the promotion of apoptosis. Therefore, if apoptosis does not occur, cancer cells proliferate on a large scale.

The function of *TRAIL* can be decreased or inhibited through several different mechanisms, which include increased expression of decoy deceptors (*DcR1* and *DcR2*) that compete with death receptors (*DR4* and *DR5*) for binding with *TRAIL*; mutations in *DR4* and *DR5* or even their decreased expression; and increased expression of *TRAIL* inhibitors that are also called antiapoptotic proteins [39,40].

In this study, we considered that miR-106b-5p could be one of these antiapoptotic genes that interfere in the *TRAIL* apoptosis pathway. Unlike this gene, miR-106b-5p had an increased expression in the Cancer group, the same that presented low expression of *TRAIL*. Although the expression of these genes presents important statistical differences in the Cancer group, we confirmed that miR-106b-5p does not interfere directly in *TRAIL* but in genes activated from *TRAIL* signaling. Therefore, the establishment of cancer is probably due to the interference of miR-106b-5p in another gene in the apoptosis pathway other than *TRAIL*. MicroRNAs regulate functions for gene expression and they can be involved in carcinogenesis depending on their target [41]. In a more refined analysis, the miRWalk databases were used to determine validated targets miR-106b-5p genes involved in gastric carcinogenesis and 14 genes from this pathway were found: APC (regulator of the WNT signaling pathway), CCND1 (cyclin D1) CDKN1A (cyclin-dependent kinase inhibitor 1), E2F1, E2F2, E2F3 (transcription factor 1, 2, and 3, respectively), FZD9 (frizzled class receptor 9), MAPK1 (mitogen-activated protein kinase 1), MYC (proto-oncogene BHLH transcription factor), RB1 (transcriptional corepressor 1), SMAD4 (SMAD family member 4), TCF7L2 (transcription factor 7-like 2), TGFBR2 (transforming growth factor β receptor 2), and TP53 (tumor protein p53). Considering the main functions described in the process of carcinogenesis, these genes are involved in cell cycle regulation and tumor progression, cell growth, adhesion and migration, proliferation, differentiation, genome stability, DNA repair, and apoptosis [42,43,44,45,46,47,48,49].

Studies have demonstrated that miR-106b-5p is related to cell proliferation, invasion, and metastasis in the esophagus, gastric, breast, hepatic, renal, and lung cancer [50,51,52]. Fan et al. [41] concluded in their work that miR-106b-5p expression was increased in cervical cancer, and by suppressing this microRNA, cancer could be inhibited through apoptosis of the malignant cells. Similarly, Yin et al. [50] found an upregulation of miR-106b-5p in prostate cancer and concluded that inhibiting this microRNA could be an effective therapy against this cancer. We suggest that miR-106b-5p may act as an oncogene in gastric cancer, considering its high expression in the Cancer group and the ability to target genes in the apoptosis pathway and control them.

The results correlating *TRAIL* expression and *H. pylori* indicated that this bacterium does not influence this gene expression. Both results that considered or not the presence of *H. pylori* were similar and showed no difference for *TRAIL* expression, which was still decreased. On the other hand, *H. pylori* does influence miR-106b-5p expression, which was decreased in groups that were positives for the presence of the bacterium (Gastritis Pos. and Cancer Pos.).

Ye et al. [53] suggest that *H. pylori*’s lipopolysaccharides (LPS), a virulence factor of this bacterium, activate the toll-like receptor 4 (TLR4) signaling pathway, which is capable of downregulating the expression of miR-106b-5p, contributing to this miRNA-decreased expression. In addition, the authors confirm that *JAK1* and *STAT3* are target genes of miR-1006b-5p and that the *JAK1/STAT3* pathway plays a part in gastric inflammation (gastritis establishment) and carcinogenesis.

The result for the presence of *H. pylori* in the Cancer group was already expected considering that the relationship of this bacterium with the occurrence of gastric neoplasia has already been proven [54,55,56]. Considering the bacterium and *TRAIL*, these two parameters are not related to each other on the onset of cancer. Taking the miR-106b-5p, this gene is able to start the oncogenic process due to its ability to control some genes that regulate the apoptosis, which is a defense mechanism against tumoral processes; we noticed that *H. pylori* is able to decrease this microRNA expression, therefore it might be able to avoid gastric cancer onset by this gene, but still, the presence of this bacterium by itself could be enough to promote the oncogenic process.

When the *TRAIL* gene was tested in silico considering samples of gastric cancer, was verified a decreased expression, in at least one database, confirming our results. The dysregulation of the *TRAIL* gene and in miR-106b-5p may suggest a double hit in an important pathway as apoptosis and gastric cancer.

## 5. Conclusions

Together, our results demonstrate that *H. pylori* is a risk to the development of gastric disease, a result that is already described in the literature. Furthermore, we verified a decrease of TRAIL gene expression in patients with gastric cancer, considering or not the presence of *H. pylori*. Interestingly, our results suggest that the expression of miR-106b-5p behaves differently in patients with gastritis and gastric cancer since we verified an increase of expression of miR-106b-5p in the Gastric Cancer group and a decrease of expression in the Gastritis group. An increase of expression of miR-106b-5p also found in patients with gastric cancer negative to *H. pylori*; on the other hand, an intense decrease of expression was found in patients with gastritis positive to *H. pylori*, suggesting the possible involvement of this bacterium in the control of miR-106b-5p expression.

## Figures and Tables

**Table 1:** Patient and group information.

Groups	Control	Gastritis	Cancer	Patients Ethnic Origins (%)
Male (%)	22 (36.7)	53 (40.16)	30 (57.7)	European (95%)
Female (%)	38 (63.3)	79 (59.84)	22 (42.3)	Japanese (2.5%)
Total (%)	60 (100)	132 (100)	52 (100)	African (2.5%)
Mean Age ± SD	55 ± 15.6	54 ± 16	53 ± 10	

**Table 2:** *H. pylori* detected in the Control, Gastritis, and Cancer groups.

	Control (%)	Gastritis (%)	Cancer (%)	Total (%)
***H. pylori +***	11 (18.3)	54 (40.9)	38 (73.1)	103 (42.2)
***H. pylori −***	49 (81.7)	78 (59.1)	14 (26.9)	141 (57.8)
**OR (95% CI)**		3.08 (1.45–6.64)	12.09 (4.73–28.7)	
***p***		0.0028 *	<0.0001 *	
**Total**	60 (100)	132 (100)	52 (100)	244 (100)

* statistically significant.

**Table 3:** RQ mean for the groups considered in miR-106b-5p mRNA expression and *p-value* results for comparison between the groups.

*Group (RQ Mean)*	*Compared Groups*	*p-Value*
Control (0.9950)	Control vs. Gastritis	0.0305 *^A^
Gastritis (0.9200)	Control vs. Cancer	0.0307 *^B^
Cancer (1.080)	Gastritis vs. Cancer	0.0004 *^C^
Control Neg. (0.9900)	Control Neg. vs. Control Pos.	0.4559
Control Pos. (0.9900)	Gastritis Neg. vs. Gastritis Pos.	<0.0001 *^D^
Gastritis Neg. (1.020)	Cancer Neg. vs. Cancer Pos.	0.0192 *^E^
Gastritis Pos. (0.7800)	Control Neg. vs. Gastritis Neg.	0.4068
Cancer Neg. (1.350)	Control Neg. vs. Gastritis Pos.	<0.0001 *^F^
Cancer Pos. (1.000)	Control Neg. vs. Cancer Neg.	0.0001 *^G^
	Control Neg. vs. Cancer Pos.	0.5370

* statistically significant. The *p*-values (*A–*G) refer to *p*-values of Figure 4.

**Table 4:** Screening DEGs in gastric cancer.

DEGs	Genes
Upregulated	MT1E, C1orf132, SST, GPX3, MAL, ATP4B, RNASE1, SCUBE2, C16orf89, CHGA
Downregulated	IVNS1ABP, THY1, SMARCA4, HAVCR2, INHBA, GTPBP4, TMEM158, MFSD12, COL18A1, PLA2G7, SSR2, COL1A1, CTHRC1, SERPINH1, IFI30, OLFML2B, SOD2, SPARC, FAM20C, MFSD13A, MYO1B, C1orf112, AGPAT4, KIF26B, S100A10, SULF1, UCK2, CENPF, LOX, PMEPA1, NCR3LG1, CTSA, ANGPT2, CENPN, SPON2, NUF2, APOC1, PGM2L1, FAP, COL12A1, KIF18B, ARHGAP39, CKS1B, IGF2BP3, FRP4, MMP3, LRP8, CLDN4, SLC4A11, LINC01296, EPHB2

## Supplementary Materials

The following are available online at https://www.mdpi.com/2073-4425/11/4/393/s1, Figure S1: 2% agarose gel stained with ethidium bromide showing H. pylori PCR products (150bp) used to identify H. pylori in gastric biopsy samples from patients with gastrites. “Slots C+ and C-” Positive and Negative Controls, respectively. “Slot M” Ladder Marker 100 bp (Invitrogen). Water was used as negative control; as positive control, H. pylori DNA from culture was used. The band of approximately 1200bp is a non-specific band, not associated with H. pylori, Figure S2: agarose gel stained with ethidium bromide showing H. pylori PCR products (150bp) used to identify H. pylori in gastric biopsy samples from patients with gastric cancer. “Slots C+ and C-” Positive and Negative Controls, respectively. “Slot M” Ladder Marker 100 bp (Invitrogen). Water was used as negative control; as positive control, H. pylori DNA from culture was used. The band of approximately 1200bp is a non-specific band, not associated with H. pylori, Figure S3: 2% agarose gel stained with ethidium bromide showing H. pylori PCR products (150bp) used to identify H. pylori in gastric biopsy samples from patients with normal gastric tissue. “Slots C+ and C-” Positive and Negative Controls, respectively. “Slot M” Ladder Marker 100 bp (Invitrogen). Water was used as negative control; as positive control, H. pylori DNA from culture was used. The band of approximately 1200bp is a non-specific band, not associated with H. pylori, Figure S4: 2% agarose gel stained with ethidium bromide showing H. pylori PCR products (150bp) used to identify H. pylori in gastric biopsy samples from patients with normal gastric tissue (Slots 1-8) and gastrites (Slots 9-17). “Slots C+ and C-” Positive and Negative Controls, respectively. “Slot M” Ladder Marker 100 bp (Invitrogen). Water was used as negative control; as positive control, H. pylori DNA from culture was used. The band of approximately 1200bp is a non-specific band, not associated with H. pylori.
