# Supplementary material for: Analysis of Gene Expression of miRNA-106b-5p and TRAIL in the Apoptosis Pathway in Gastric Cancer
Source: Genes (Basel). 2020 Apr 5;11(4):393. doi: 10.3390/genes11040393 (PMC7230378; doi:10.3390/genes11040393)

Supplementary Material

Figure S1. – 2% agarose gel stained with ethidium bromide showing *H. pylori* PCR products (150bp) used to identify *H. pylori* in gastric biopsy samples from patients with gastrites. "Slots C+ and C-" Positive and Negative Controls, respectively. "Slot M" Ladder Marker 100 bp (Invitrogen). Water was used as negative control; as positive control, *H. pylori* DNA from culture was used. The band of approximately 1200bp is a non-specific band, not associated with *H. pylori*.

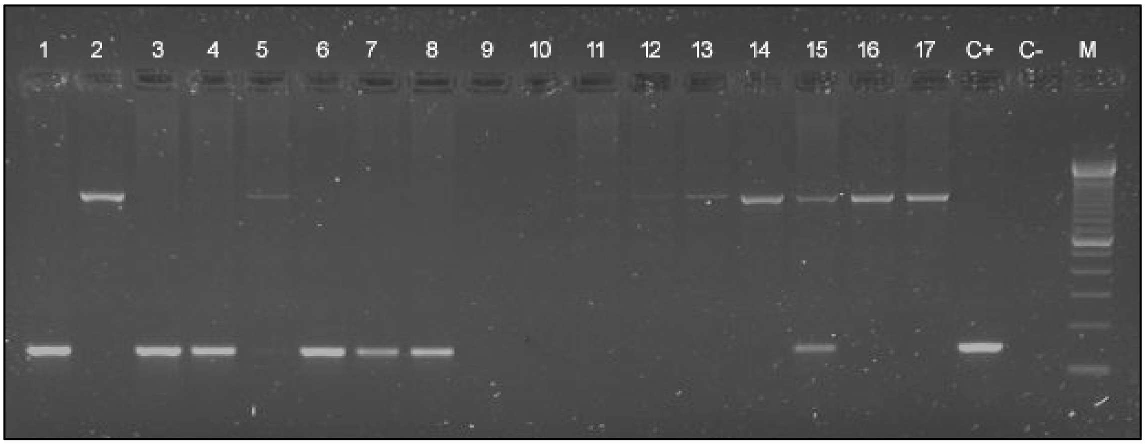

Figure S2. – 2% agarose gel stained with ethidium bromide showing *H. pylori* PCR products (150bp) used to identify *H. pylori* in gastric biopsy samples from patients with gastric cancer. "Slots C+ and C-" Positive and Negative Controls, respectively. "Slot M" Ladder Marker 100 bp (Invitrogen). Water was used as negative control; as positive control, *H. pylori* DNA from culture was used. The band of approximately 1200bp is a non-specific band, not associated with *H. pylori*.

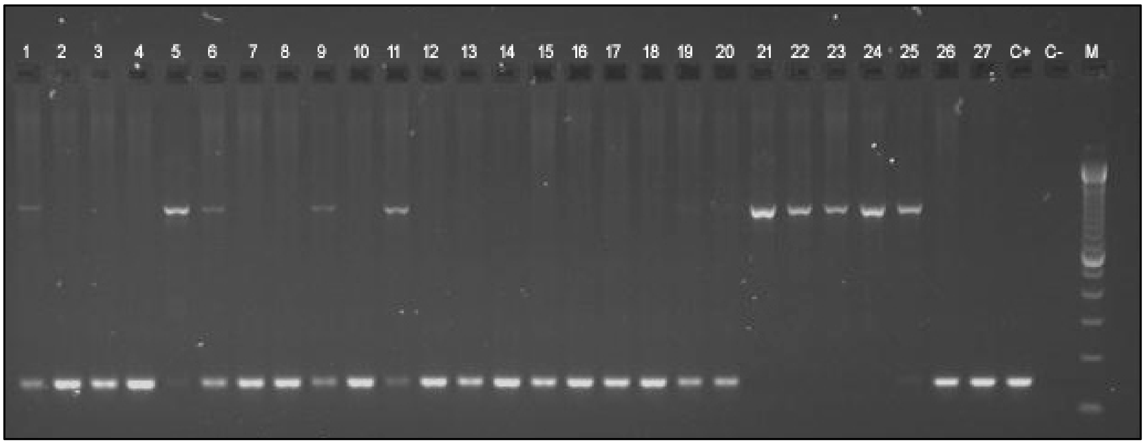

Figure S3. – 2% agarose gel stained with ethidium bromide showing *H. pylori* PCR products (150bp) used to identify *H. pylori* in gastric biopsy samples from patients with normal gastric tissue. “Slots C+ and C-” Positive and Negative Controls, respectively. “Slot M” Ladder Marker 100 bp (Invitrogen). Water was used as negative control; as positive control, *H. pylori* DNA from culture was used. The band of approximately 1200bp is a non-specific band, not associated with *H. pylori*.

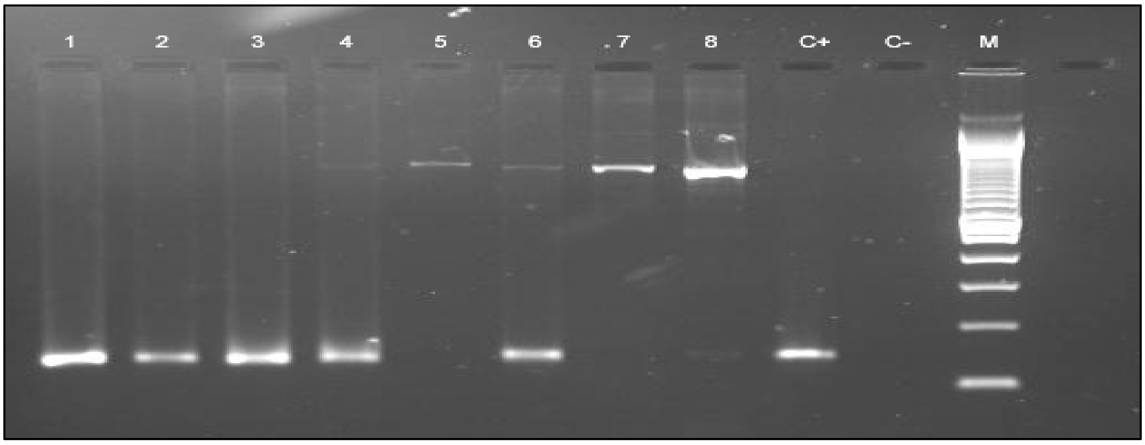

Figure S4. – 2% agarose gel stained with ethidium bromide showing *H. pylori* PCR products (150bp) used to identify *H. pylori* in gastric biopsy samples from patients with normal gastric tissue (*Slots 1-8*) and gastrites (*Slots 9-17*). "Slots C+ and C-" Positive and Negative Controls, respectively. "Slot M" Ladder Marker 100 bp (Invitrogen). Water was used as negative control; as positive control, *H. pylori* DNA from culture was used. The band of approximately 1200bp is a non-specific band, not associated with *H. pylori*.

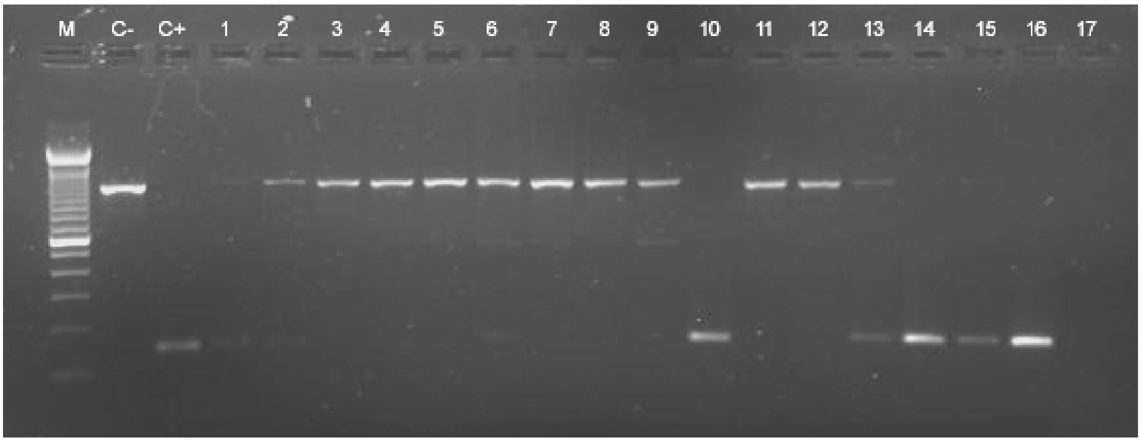

Supplement: Supplementary file 1 [file genes-11-00393-s001.pdf]
